# Operative Repair of Type A Aortic Dissection in Jehovah’s Witnesses: Insights From a Case Series

**DOI:** 10.1016/j.atssr.2023.11.007

**Published:** 2023-11-23

**Authors:** James Lee West, Clifton Lewis, Sasha Still, Kyle Eudailey

**Affiliations:** 1 Division of Cardiothoracic Surgery, Department of Surgery, University of Alabama at Birmingham, Birmingham, Alabama

## Abstract

**Background:**

Jehovah’s Witnesses are known to refuse allogeneic blood products. Consequently, many surgeons will decline to perform aortic surgery on this patient group, given their higher risk. We present here a case series describing operative repair of type A aortic dissection without the use of allogeneic blood products.

**Methods:**

From 2018 to 2021, 6 Jehovah’s Witness patients underwent open repair of type A aortic dissection. We reviewed preoperative characteristics, diagnostic workup, operative technique, and postoperative outcomes. We also looked specifically at use of autologous whole blood, Cell Saver (Haemonetics) transfusion, synthetic clotting factors (recombinant factor VIIa), and albumin and prothrombin complex concentrate when allowed.

**Results:**

Modified hemiarch replacement was performed with the branched single-anastomosis frozen elephant trunk repair (B-SAFER) technique in 4 patients; 2 patients received a standard hemiarch replacement. Aortic valve replacement was performed in 2 patients; root replacement was performed in 1 patient. There were no immediate postoperative coronary complications, strokes, or instances of renal failure. Average decrease in hemoglobin concentration after surgery was 2.3 ± 1.5 g/dL (mean ± SD). Thirty-day mortality was 0. Five of 6 patients survived to discharge, and all survivors to discharge were still alive at the time of submission.

**Conclusions:**

Bloodless aortic dissection repair in these rare patients can be performed safely in specialized centers using the techniques described. Furthermore, we believe that these techniques can be applied to the general population of patients undergoing emergent cardiac operation to avoid allogeneic transfusions.

**In Short**

▪ Here we present a series demonstrating excellent short-term outcomes in operative repair of acute type A dissection in 6 Jehovah’s Witness patients.
▪ This series provides technical and procedural insights to guide bloodless surgery in patients with type A aortic dissection and optimal operative treatment in this population of difficult-to-treat patients.

Acute aortic surgery almost universally involves transfusion of sometimes large amounts of allogeneic blood products. However, Jehovah’s Witnesses are known to refuse use of allogeneic blood products for religious reasons. Consequently, many surgeons will decline to perform aortic surgery on this patient group, given their relatively higher risk-benefit ratio. We present here a case series describing operative repair of type A aortic dissection (TAAD) without the use of allogeneic blood products in Jehovah’s Witnesses.

## Patients and Methods

### Patients

From 2018 to 2021, a total of 6 Jehovah’s Witness patients underwent open repair of TAAD without any transfusion of allogeneic blood products. Patients’ demographics and preoperative characteristics are summarized in Table 1. All patients were transferred from outside facilities where they were denied surgery because of refusal of blood products. All the acute TAADs underwent surgery within 72 hours of diagnosis at outside facilities. In all cases, informed consent was obtained. This included a detailed discussion about the risk of emergent cardiac surgery without the use of allogeneic blood products and consent for use of autologous whole blood, Cell Saver (Haemonetics) transfusion, synthetic clotting factors (recombinant factor VIIa), and albumin and prothrombin complex concentrate (PCC) when allowed.

**Table 1:** Patients’ Demographics and Preoperative Characteristics

Patient	Age (y)	Sex	Aortic Disease	DeBakey Type	Origin of Dissection	Extent of Dissection	Status	Time to OR (d)	Evidence of Malperfusion	Comorbidities	BMI (kg/m^2^)
1	62	Female	Subacute type A dissection	I	Zone 0 (close to RCA ostium)	Right common iliac (zone 10)	Elective	22	No	Hypertension, atrial fibrillation, diabetes	27.5
2	57	Female	Acute type A dissection	I	Zone 0 (STJ)	Descending thoracic aorta (zone 5)	Emergent	2	No	Hypertension, hyperlipidemia, morbid obesity	35.6
3	76	Male	Acute type A dissection	I	Zone 0 (root)	Bilateral common iliac (zone 10)	Emergent	1	No	Hypertension	25.1
4	71	Male	Acute type A dissection	I	Zone 1	Left common and external iliac (zone 11)	Emergent	2	No	Hypertension, polycythemia, obstructive sleep apnea	22.2
5	64	Male	Acute type A dissection	II	Zone 0	Zone 1	Emergent	1	No	Hyperlipidemia, rheumatoid arthritis	38.6
6	62	Male	Acute type A dissection	I	Zone 0	Left femoral	Emergent	2	No	Hypertension	28.7

BMI, body mass index; OR, operating room; RCA, right coronary artery; STJ, sinutubular junction.

### Operative Technique

All patients received a central venous line and bilateral radial arterial lines. Before incision, 2 units (600 mL) of autologous normovolemic hemodilution (ANH) whole blood was withdrawn and drained into a citrate-containing bag connected to the central line that remained in circuit with the body and thereby fulfilled religious requirements.

Central aortic and venous cannulation was undertaken in all patients. Tranexamic acid was given before cardiopulmonary bypass to prevent fibrinolysis. Weight-based heparin was given for goal activated clotting time >450 seconds. Care was taken to limit the size of the bypass circuit, and autologous priming was performed to limit dilution.

The head was packed with ice, and cardiopulmonary bypass was initiated with cooling to 28 °C in 3 patients and 30 °C in the other 3 patients. Propofol was given before circulatory arrest, and electroencephalogram silence was confirmed on bispectral index. Once target temperature was achieved, the patient was placed in steep Trendelenburg position, and the cardiopulmonary bypass circuit was stopped. The patient was drained to minimize blood loss. Aortic cannulas were then removed, and the aortic arch was resected. Bilateral selective antegrade cerebral perfusion was used in all patients. Cannulas were placed in the innominate artery and the left common carotid artery. Antegrade perfusion was performed at a rate of 8 to 10 mL/kg per minute, and noninvasive cerebral saturations were monitored. Retrograde and direct ostial antegrade del Nido cardioplegia was used.

In 4 patients, modified hemiarch replacement was performed with the addition of the branched single-anastomosis frozen elephant trunk repair (B-SAFER) technique, which included a frozen elephant trunk and left subclavian fenestration and stenting.^1^ A standard hemiarch replacement was performed in 2 patients. Three patients underwent concomitant aortic valve replacement.

At the time of discontinuation of cardiopulmonary bypass, protamine was given to reverse heparin. The patients received autologous transfusion of 600 mL of previously drawn blood from the central line. Cell Saver blood, which always remained in circuit with the patient, was also used.

## Results

Table 2 summarizes intraoperative data, cardiopulmonary bypass times, and clotting factors and other medications given for hemostasis. Mean cardiopulmonary bypass time was 135.8 ± 17.9 minutes (mean ± SD; range, 114-166 minutes). Mean circulatory arrest time was 15.1 ± 11.4 minutes (6-35 minutes). Mean preoperative hematocrit was 36.3% ± 4.8%.

**Table 2:** Individual Intraoperative Data

Patient	Operation	Cannulation	Factors and Medications Given	Total OR Time (min)	CPB Time (min)	Selective Antegrade Perfusion Time (min)	Circulatory Arrest Time (min)	Estimated Blood Loss (mL)	Cooling Temperature (°C)	Hematocrit (%)	
										Preoperative	Postoperative
1	Total arch replacement, frozen elephant trunk with left subclavian stent, aortic valve replacement	Central	Recombinant factor VIIa Desmopressin	483	166	27	35	300	28	37	33
2	Hemiarch replacement	Central	PCC	259	136	22	6	200	28	32	24
3	Total arch replacement, frozen elephant trunk, aortic valve replacement	Central	PCC	310	135	30	7	200	30	32	24
4	Ascending aorta replacement, hemiarch repair, frozen elephant trunk with left subclavian stent	Central	None given	333	122	25	6	200	30	43	30
5	Hemiarch, frozen elephant trunk with left subclavian stent	Central	PCC	246	114	10	17	300	30	41	35
6	Hemiarch, root replacement	Central	PCC	268	142	16	20	300	28	33	25

CPB, cardiopulmonary bypass; OR, operating room; PCC, prothrombin complex concentrate.

Table 3 summarizes postoperative results. Mean postoperative decrease in hematocrit was 7.8% ± 3.3% (4%-13%). Intensive care length of stay was 3.2 ± 0.4 days (3-4 days). Mean hospital length of stay was 12.8 ± 11.1 days (5-35 days). There were no immediate postoperative coronary complications, strokes, or instances of renal failure. The 30-day mortality was 0. There was 1 in-hospital mortality on postoperative day 35, which also constituted the only 1-year mortality (patient 1, subacute dissection). This patient presented with vocal cord paralysis and aspirated postoperatively, leading to the development of aspiration pneumonia followed by sepsis.

**Table 3:** Postoperative Data

Patient	Hemoglobin (g/dL) Postoperative (preoperative) POD 1		Extubation Time (h)	ICU Length of Stay (d)	Hospital Length of Stay (d)	30-Day Mortality	1-Year Mortality	Discharge Creatinine (mg/dL)	Stroke	MI	Reentry
1	11.2 (11)	10	9	2 (readmitted to ICU POD 34)	35	0	POD 35	N/A	0	0	0
2	8.2 (10.3)	8.8	33	3	8	0	0	0.56	0	0	0
3	8.2 (10.9)	7.4	5	3	9	0	0	1.34	0	0	0
4	10.3 (14.6)	10.3	16	4	8	0	0	1.5	0	0	0
5	11.3 (13.5)	10	6	3	5	0	0	1.2	0	0	0
6	8.3 (11.0)	8.0	7	3	12	0	N/A	2	0	0	0

ICU, intensive care unit; MI, myocardial infarction; N/A, not applicable; POD, postoperative day.

Follow-up computed tomography scans showed a mix of favorable remodeling and residual dissection disease. The Figure shows an illustrative example of favorable remodeling in patient 3 at 1 week postoperatively before discharge.

**Figure:**
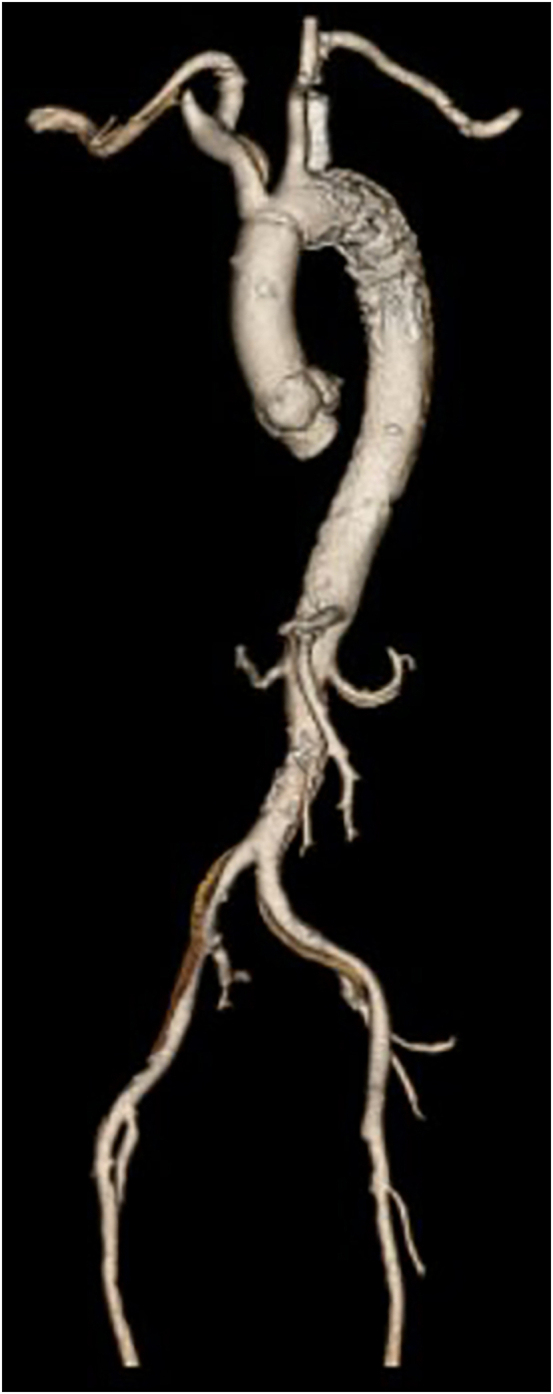
Illustrative example of favorable remodeling, patient 3.

## Comment

Patients requiring cardiac surgery who refuse blood products pose an ethical dilemma for surgeons. Multiple studies at specialized centers have shown that elective cardiac surgeries without blood transfusions do not have increased mortality compared with patients who are able to receive blood products.^2^, ^3^, ^4^ However, the data regarding bloodless surgery in emergent TAAD repair are limited. Here we present a series to date demonstrating excellent short-term outcomes in 6 Jehovah’s Witness patients.

The concept of conservation is of paramount importance throughout the entirety of the hospitalization. Whereas there is literature to support the use of erythropoiesis-stimulating agents in the preoperative setting,^4^ this is rarely useful, given the emergent nature of dissection. We thus focus on conservation by limiting blood draws preoperatively, intraoperatively, and postoperatively. Once in the operating room, all patients underwent ANH protocol in accordance with their religious beliefs. ANH is a proven technique in minimizing transfusion requirements in circulatory arrest.^5^ During the operation, care was taken to constantly achieve hemostasis. Cardiopulmonary bypass and autologous blood recovery systems may be used if the blood remains in constant circuit with the patient.^6^ In addition, limitation of the bypass circuit size and arterial and venous autologous priming prevent hemodilution. In all cases, we used moderate hypothermia, between 28 and 30 °C, and bilateral selective antegrade perfusion, which has been proven to be a safe technique in arch surgery.^7^ We believe limiting hypothermia significantly decreases the associated coagulopathy without sacrificing neuroprotection.

Technically, we prefer to employ the B-SAFER modified frozen elephant trunk technique with fenestration and left subclavian stenting.^1^ This was performed in 4 of the 6 patients. We believe this allows the maximal amount of treatment of the aortic disease with minimized circulatory arrest time. We anecdotally believe this helps with hemostasis and limits postoperative malperfusion through stabilization of the true lumen. One patient received a standard hemiarch based on the anatomy of the dissection and tear, which was a DeBakey type I with thrombosis of the false lumen, the arch, and the descending aorta. One patient received a hemiarch in the setting of a type II dissection. Root replacement was avoided in all but 1 patient, given the increased bleeding risk.

Most Jehovah’s Witness patients are unable to accept whole blood, predonated autologous blood, packed red blood cells, platelets, or plasma. There are “matter of conscience” products that may be accepted, depending on individual preference; these are clotting factors, PCC, and albumin. Recombinant factor VIIa is typically acceptable. We make sure to have clear conversations with patients preoperatively to understand what is at our disposal for hemostasis. If acceptable, we prefer to use PCC after protamine reversal and delivery of ANH blood. PCC use in cardiac surgery in meta-analysis has been proven to reduce transfusion requirements, with no clear additional risk of stroke,^8^ whereas the literature suggests an increased risk of stroke in the setting of factor VII use.^9^ Patients also received desmopressin, which is thought to aid with bleeding from platelet dysfunction after major surgery.^10^

We present this series to demonstrate the techniques available in treating this morbid disease in a population of difficult-to-treat patients. As is true of any surgery, benefits, risks, and alternatives must be discussed in depth with the patient during the process of informed consent. We ultimately believe bloodless aortic dissection repair in these rare patients can be performed safely in specialized centers using the techniques described. Furthermore, we believe that these techniques can be applied to the general population of patients undergoing emergent cardiac operation to avoid allogeneic transfusions as these have been associated with poorer short-term and long-term outcomes in cardiac surgery.
